# In situ characterisation and manipulation of biological systems with Chi.Bio

**DOI:** 10.1371/journal.pbio.3000794

**Published:** 2020-07-30

**Authors:** Harrison Steel, Robert Habgood, Ciarán L. Kelly, Antonis Papachristodoulou

**Affiliations:** 1 Department of Engineering Science, University of Oxford, Oxford, United Kingdom; 2 Department of Applied Sciences, Faculty of Health and Life Sciences, Northumbria University, Newcastle upon Tyne, United Kingdom; University of Sussex, UNITED KINGDOM

## Abstract

The precision and repeatability of in vivo biological studies is predicated upon methods for isolating a targeted subsystem from external sources of noise and variability. However, in many experimental frameworks, this is made challenging by nonstatic environments during host cell growth, as well as variability introduced by manual sampling and measurement protocols. To address these challenges, we developed Chi.Bio, a parallelised open-source platform that represents a new experimental paradigm in which all measurement and control actions can be applied to a bulk culture in situ. In addition to continuous-culturing capabilities, it incorporates tunable light outputs, spectrometry, and advanced automation features. We demonstrate its application to studies of cell growth and biofilm formation, automated in silico control of optogenetic systems, and readout of multiple orthogonal fluorescent proteins in situ. By integrating precise measurement and actuation hardware into a single low-cost platform, Chi.Bio facilitates novel experimental methods for synthetic, systems, and evolutionary biology and broadens access to cutting-edge research capabilities.

## Abstract 2

The precision and repeatability of biological studies are predicated upon methods for isolating a targeted subsystem from external sources of noise and variability. This article presents a low-cost, open-source culture platform, Chi.Bio, which allows biologists to automate experimental protocols with unparalleled precision.

## Introduction

Experimental methods for precise characterisation and manipulation of in vivo biological systems are critical to their study [1]. Difficulties often arise because of a lack of control over the conditions experienced by cells prior to and during an experiment, which makes isolating a cellular subsystem's behaviour from that of its host challenging [2, 3]. For example, in widely used batch culture methods [4, 5], a cell's external chemical environment varies significantly as a culture grows [6], leading to physiological changes in factors including growth phase and resource availability [2]. Additional variability is then introduced at the point of measurement, which often requires manual handling of samples and interaction between multiple pieces of hardware that are themselves variable between conditions and laboratories [7, 8]. These weaknesses of typical experimental techniques have limited scientists' capabilities and led to a crisis of reproducibility in biology [9].

To improve the robustness of biological data, an ideal experimental setup would provide a controlled, static environment in which culture parameters such as nutrient availability and temperature are regulated [10] and would perform frequent and accurate measurements in situ. This can be partially achieved using continuous culture devices such as a turbidostat, which dilutes cells during growth to maintain a constant optical density (OD). In recent years, a number of turbidostat platforms have been developed and are beginning to find widespread applications in systems, synthetic, and evolutionary biology [11–19]. There has also been significant development of open-source platforms for optogenetics [20, 21], which have been used to implement real-time measurement [22] and feedback control [23] and to demonstrate orthogonal regulation of multiplexed optogenetic regulators [24]. However, in many cases open-source platforms have not been easy to build/obtain, require interfacing with external hardware (such as incubators), are inflexible for applications beyond their designed purpose, and only provide a limited subset of the in situ measurement and actuation capabilities (such as fluorescence spectrometry or optogenetic actuation) that are fundamental to many experimental studies.

**Fig 1:**
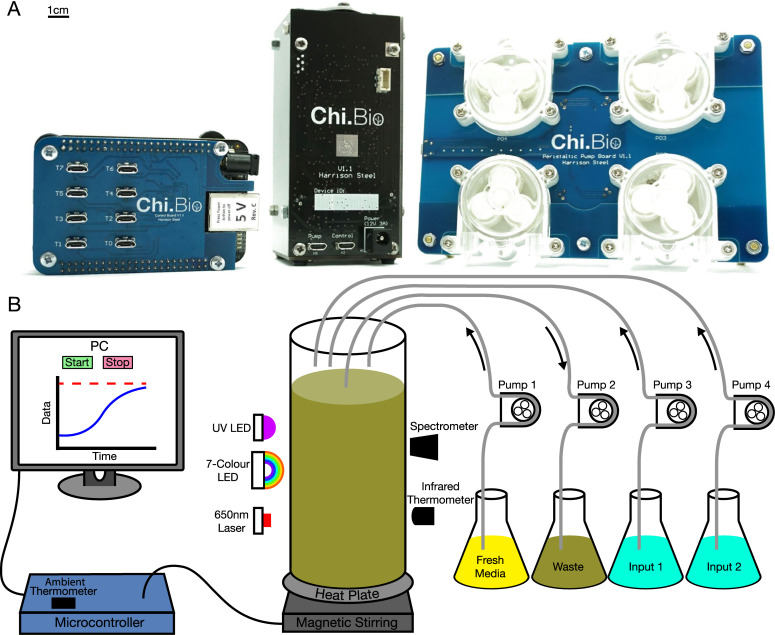
The Chi.Bio platform. **(a)** The platform comprises a control computer (left), main reactor (centre), and peristaltic pump board (right). It is open source and can be constructed for approximately $300 using only PCBs and off-the-shelf components. Scale bar indicates 1 cm, giving the main reactor dimensions of 11.5 × 5.3 × 5.3 cm. **(b)** Schematic of subsystems and interconnections. A lab computer or network connects to the control computer, which runs the platform's operating system and can interface with up to eight reactor/pump pairs in parallel. Each reactor has a 12- to 25-mL working volume and contains a range of measurement and actuation tools for precise in situ manipulation of biological systems. These include a UV LED, a 650-nm laser (for OD measurement), seven-colour LEDs in the visible range (for optogenetics and fluorescence measurement), and a spectrometer. An infrared thermometer and heat plate are used to regulate temperature, and the culture is agitated using magnetic stirring. Each reactor has a modular pump board with four direction- and speed-controllable peristaltic pumps. For a detailed descriptions of each hardware subsystem, see S1A–S1D Data. LED, light-emitting diode; OD, optical density; PC, personal computer; PCB, printed circuit board.

To address these challenges, we developed Chi.Bio, a parallelised all-in-one platform for automated characterisation and manipulation of biological systems. It is open source and can be built from printed circuit boards (PCBs) and off-the-shelf components for approximately $300 per device. The platform comprises three primary components (Fig 1A): a control computer, main reactor, and pump board. The control computer can interface with up to eight reactor/pump pairs in parallel, allowing independent experiments to be run on each. It also hosts the platform's operating system, which provides an easy-to-use web interface for real-time control and monitoring of ongoing experiments. The main reactor contains most of the platform's measurement and actuation subsystems (Fig 1B), which operate on standard 30-mL flat-bottom screw-top laboratory test tubes (with a 12- to 25-mL working volume). All measurement and actuation systems (with the exception of the heat plate) are noncontact, minimising sterilisation challenges and allowing test tubes to be hot swapped during operation. Each reactor can accept up to four liquid in-/outflow tubes, which are driven by peristaltic pumps housed in the reactor's dedicated pump board. The platform as a whole is entirely modular (the three components interconnect via micro-USB cables), allowing it to be tailored to a wide variety of experimental configurations.

## Results

### Hardware subsystems

**Fig 2:**
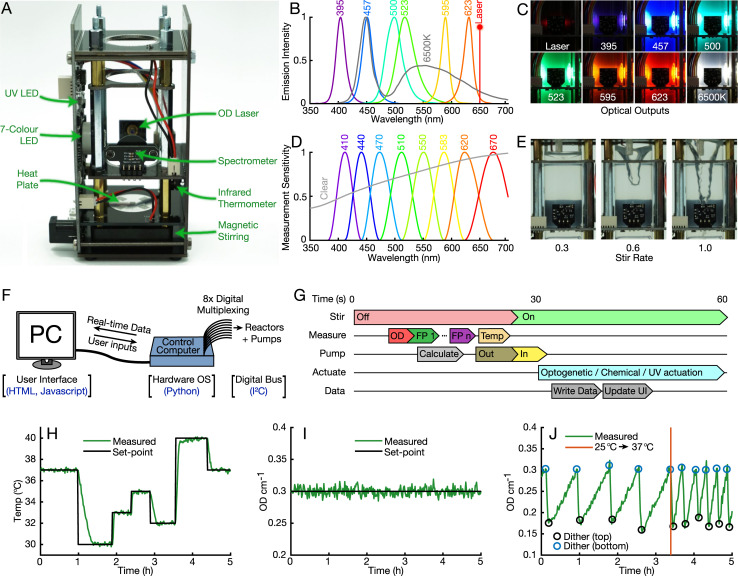
Hardware subsystems, software, and automation. **(a)** The main reactor with sides removed and subsystems labelled. The vertical PCB on the left hosts driving circuitry for many subsystems, as well as power regulation and filtering. **(b)** Emission spectra of the visible optical outputs in the device (UV LED peak is at 280 nm). **(c)** Images of optical outputs, with laser set to 50% and LEDs to 5% intensity. LEDs are focused and perpendicular to the spectrometer to maximise faint fluorescence signals. **(d)** Measurement filter bands of the platform's spectrometer. **(e)** Magnetic stirring provides a powerful vortex when set to a high rate. **(f)** Software architecture, which packages multiplexed low-level commands (digital communications following I^2^C standard) into an easy-to-use web interface accessed from a connected PC or network. **(g)** A typical 60-second experimental automation cycle. Initially stirring is disabled so liquid can settle, reducing noise in measurements and providing a flat surface for removal of waste media. **(h)** Media temperature (‘Temp’) controlled to follow a predefined path over 5 hours. Heat input is provided by the heat plate; cooling is passive. **(i)** The OD of *Escherichia coli* in exponential growth phase, maintained within approximately 2% of its set-point. **(j)** OD can be set to follow a dithered waveform (with cells rapidly diluted to a lower OD value whenever the upper limit is reached) to accurately measure growth [27]. Following a change in temperature set-point from 25°C to 37°C growth accelerates significantly. FP, fluorescent protein; LED, light-emitting diode; OD, optical density; OS, operating system; PC, personal computer; PCB, printed circuit board.

Experimental techniques that exploit interactions between light and life, such as light-sensitive proteins, optogenetics, and fluorescent reporters, are ubiquitous in biological research [25, 26]. Chi.Bio contains an array of optical outputs and sensors to support these techniques (Fig 2A). A 650-nm laser is used for OD measurement (calibrated against a Spectrophotometer, S1E Data) and is driven by an analogue feedback circuit to provide stable, temperature-insensitive readings (S1F Data). Optogenetic actuation and excitation of fluorescent proteins employs a focused high-power seven-colour light-emitting diode (LED) with six emission bands across the visible range and a 6500K white output (Fig 2B and 2C), and a separate 280-nm UV LED is included to stress or kill cells in the growing population. Each LED has an independent current-limiting pulse-width modulation (PWM) driver, allowing its intensity to be regulated over three orders of magnitude (S1C Data), and is thermally coupled to the outside of the device to reject heat when operating at high intensity. The combined LED implementation provides optical outputs that exhibit minimal power and spectral variability between devices or environmental conditions (S1G Data).

Measurements of light intensity are performed within the device by a chip-based spectrometer with eight optical filters covering the visual range, as well as an un-filtered ‘Clear’ sensor (Fig 2D). Multiple wavelength bands can be measured simultaneously, each with electronically adjustable gain and integration time. The spectrometer is set up to perform temperature and long-term baseline calibration using a dark photodiode prior to every measurement. Typical spectrometer measurements (e.g., of fluorescence) are reported as the ratio of light intensity measured at the fluorescent protein's emission band to the total intensity of the excitation source; this ratiometric measurement mitigates the impact of differing excitation intensity between devices and spatial variations in culture density.

Culture temperature is measured noninvasively by a medical-grade infrared thermometer, which is accurate to ±0.2°C for temperatures near 37°C. There are also air temperature thermometers (±0.5°C accuracy) within the main reactor and on the control computer for monitoring the surrounding environment. Temperature change is actuated by a PCB-based heat plate, capable of heating a 20-mL culture at up to 2.0°C min^−1^. Below the heat plate is a magnetic stirring assembly built upon an off-the-shelf fan, which has an adjustable stirring rate (Fig 2E) and can be used with standard laboratory stir bars for aeration and mixing (S1H Data). The main reactor also includes an external expansion port, which provides power and a digital interface for user-built add-ons to Chi.Bio.

External to the main reactor is a pump board, which can house up to four low-cost peristaltic pumps with independently controllable speed (up to 1 mL s^−1^) and direction. Each pump transfers liquid to/from the culture test tube via standard 4.5-mm silicone laboratory tubing, which can be installed without joints from input to output to assist sterilisation. Typically, two pumps are dedicated to turbidostat functionality (one for input of fresh media, one for removal of waste), leaving the other two free for programmable mixing of media/inducers, or transfer of liquid between reactors. Detailed specifications of Chi.Bio's hardware and electrical subsystems are outlined in S1A–S1D Data, and analysis of their calibration, measurement stability, and interdevice variability is described in S1E–S1H Data.

### Software and automation

At the lowest level of Chi.Bio's open-source operating system is a multiplexed I^2^C bus for digital communications within the device (Fig 2F). Digital signals are in turn controlled by the hardware operating system (Python, S1I Data), which implements automation functions as well as data collection, processing, and storage (in.csv format). Real-time data are output periodically via a web server (accessible from a connected PC or network) that provides an easy-to-use web user interface (built in HTML/JavaScript, S1J Data). The complete software stack lets users set up, control, and monitor standard experiments entirely through their web browser, and more advanced protocols or algorithms that combine any of the platform's measurement/actuation capabilities can be easily implemented using the in-built ‘custom program’ Python framework (S1K Data).

A typical automated experiment follows a 60-second cycle (Fig 2G) during which the stirring vortex is allowed to settle prior to measurement, media/waste is added/removed, and data are processed to calculate new control inputs. This automation protocol can be adjusted in real time to change controller set-points or measurement setup, all of which are recorded throughout an experiment. Automated temperature and OD regulation are implemented using proportional-integral-derivative (PID) and model predictive control (MPC) algorithms [28]. The temperature control algorithm (S1L Data) facilitates rapid temperature changes with minimal error and set-point overshoot (Fig 2H). The OD control algorithm (S1M Data) typically maintains OD within approximately 2% of its set-point (Fig 2I) and can also be used to dither a culture's OD about a central set-point (Fig 2J) for precise measurement of growth rate at near-constant density [27].

## Application 1—Studying growth

**Fig 3:**
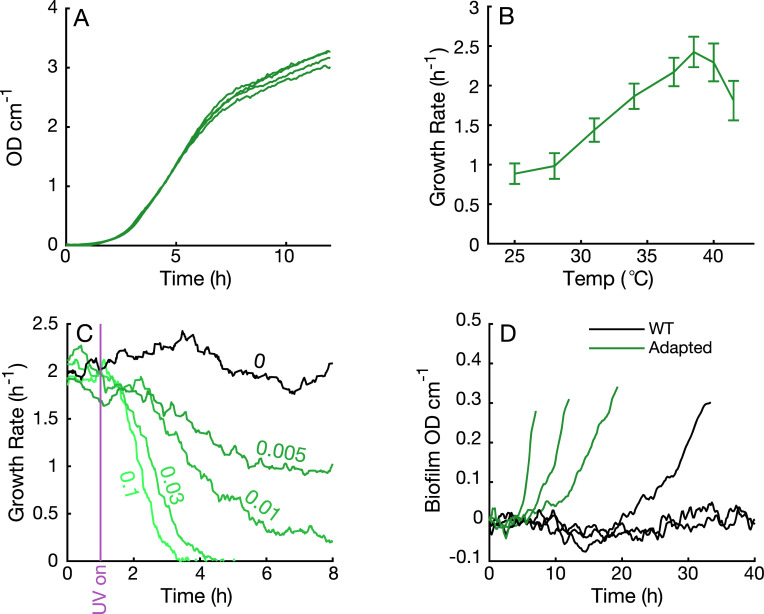
Application 1. **(a)** Growth curves of *E*. *coli* (four replicates). **(b)** Dependence of *E*. *coli* growth rate on temperature (‘Temp’); error bars represent standard deviation of growth rates measured over 5 hours at each temperature. **(c)** Measured growth rate following activation of UV source at specified power level at *t* = 1 hour. The population is able to adapt to low UV intensities and eventually returns to its initial growth rate (S1N Data). **(d)** Biofilm OD versus time for cells before and after adaptation in Chemostat mode for 120 hours, calculated as described in S1O Data. OD, optical density; WT, wild type.

A straightforward application of Chi.Bio is the study and regulation of growth in changing conditions. This can involve the collection of growth curves (Fig 3A) or dithering of OD near a set-point (as in Fig 2J) to analyse the dependence of growth on a particular parameter (e.g., temperature, Fig 3B). The UV output can be used to stress cells (or encourage mutagenesis [29]); in Fig 3C, *E*. *coli* is subjected to different UV intensities, causing a gradual reduction in growth. Chi.Bio can also be used to monitor the growth of biofilms (using the procedure outlined in S1O Data); in Fig 3D, we observe that initial adaptation to the continuous culture environment (which actively selects for biofilm-forming phenotypes [30]) leads to accelerated biofilm formation.

## Application 2—In silico feedback control

**Fig 4:**
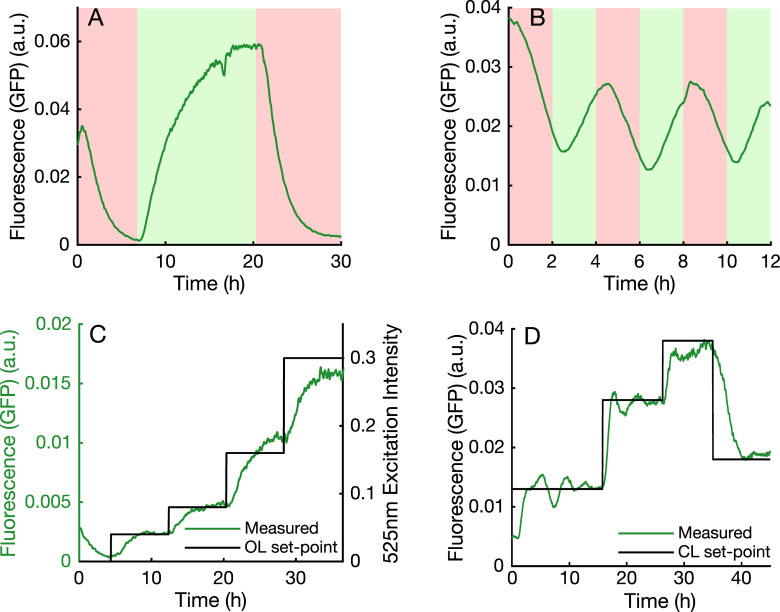
Application 2. **(a,b)** Optogenetic CcaS-CcaR system coupled to GFP expression [33], stimulated with slow- and fast-varying inputs (green/red light activate/deactivate gene expression, respectively). **(c)** Fluorescence expression (left axis) controlled by varying optogenetic excitation intensity (right axis) in OL. **(d)** Fluorescence expression regulated in CL to follow a predetermined profile, using a PI controller. a.u., arbitrary units; CL, closed loop; GFP, green fluorescent protein; OL, open loop; PI, proportional-integral.

Real-time interfacing between in silico computational elements and biological systems can facilitate novel studies [31] and optimise experimental schemes [32]. We implemented such a scheme as a ‘custom program’ in the platform, which uses the red (623 nm) and green (523 nm) LEDs as actuating inputs for the CcaS-CcaR optogenetic system coupled to green fluorescent protein (GFP) expression [33]. In Fig 4A and 4B, this system is probed with square wave inputs of varying frequency, which yield smoothed output signals (due to the dynamics of protein expression/maturation). In Fig 4C, an input of varying intensity is supplied, revealing a nonlinear dependence between fold-change of input versus output. This highlights a major challenge posed by open-loop control of biology; predicting a priori the behaviour of such complex, nonlinear systems requires accurate and implementation-specific models [5, 34], which may only be relevant in a limited range of tightly controlled environmental conditions. This can be overcome by in silico feedback control [14]: in Fig 4D, our custom program implements a control law, which updates the optogenetic excitation intensity each minute depending on the measured fluorescence, steering the culture's fluorescence to follow a complex profile without requiring a model or extensive a priori analysis of the biological system itself.

## Application 3—Multiple fluorescence outputs

**Fig 5:**
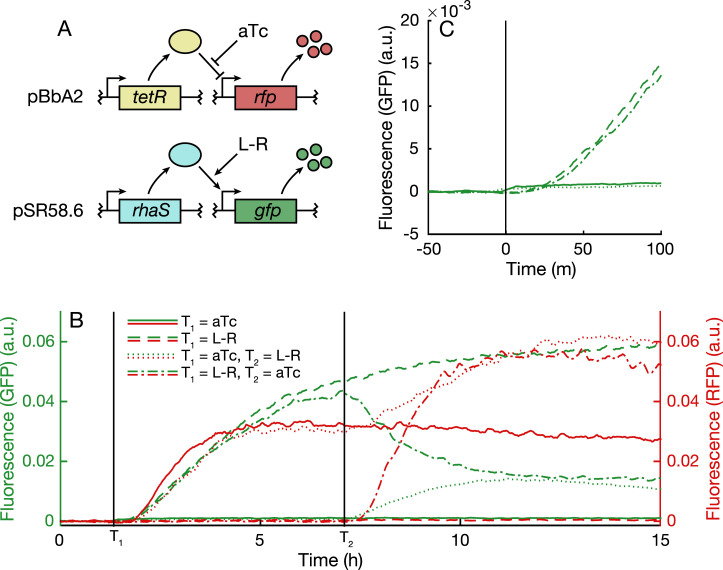
Application 3. **(a)** Two-plasmid system for inducible expression of GFP and RFP. **(b)** Fluorescence of GFP (left axis) and RFP (right axis) following induction at times *T*_1_ and *T*_2_ with indicated inducer combinations. **(c)** GFP fluorescence measured during short time period near *T*_1_. A small increase is observed in the two samples to which aTc is added due to the fluorescence of the inducer compound itself. a.u., arbitrary units; GFP, green fluorescent protein; RFP, red fluorescent protein.

Characterisation of biological systems often requires monitoring of multiple fluorescent protein–tagged outputs [35–37]. Here, we demonstrate the effectiveness of Chi.Bio for probing such a system (Fig 5A). Adding chemical inducers in different orders highlights the impact that cellular burden [35] has on its two outputs (Fig 5B): If red fluorescent protein (RFP) is induced after GFP, we observe a significant drop in GFP (due to limited resource availability for production of GFP and its activating transcription factor RhaS). However, if RFP is induced first, the subsequent induction of GFP leads to an increase in RFP expression, which we hypothesise is due to the effect of resource limitation on expression of its repressing transcription factor TetR (repeatability of this behaviour is illustrated in S1P Data). In all cases, we observe negligible cross talk between fluorescent protein–measurement channels, and our instrument's high sensitivity allows fluorescence activation to be observed within approximately 20 minutes of induction (Fig 5C, approximately the time required for fluorophore maturation).

## Discussion

The applications outlined herein represent only a fraction of Chi.Bio's potential use cases. The platform's turbidostat functionality lends its application to continuous directed evolution [38] (as demonstrated by eVOLVER [16]), which could be tuned using the UV output [29]. Direct integration of fluorescence measurement with chemical/optogenetic actuation and computational capabilities facilitates optimised experimental design [34] and the implementation of online experimental-planning algorithms [32]. Given that the only components of Chi.Bio that come into direct contact with cells are standard laboratory consumables (glass test tubes, silicone tubing, magnetic stir bars), such experiments could be run with a broad range of cell types and reagents (e.g., algae, with photosynthesis supported by the tunable LED outputs). A list of potential applications (and minor hardware modifications that can be made to enable others) is presented in S1Q Data.

The cost and resulting inaccessibility of modern scientific hardware is one of the primary obstacles to participation in cutting-edge research worldwide, particularly in small laboratories and institutions or developing nations [39]. Consequently, we have made Chi.Bio entirely open source, with schematics, code repositories, user manuals, and a public support and discussion forum available on the project's website (https://chi.bio). A single Chi.Bio device (consisting of one control computer, one reactor, and one pump board) can be assembled by hand for approximately $300 or can be purchased ready-built from a supplier of open-source scientific hardware. In the long term, we hope that Chi.Bio will provide a versatile tool for biological sciences and broaden access to cutting-edge research capabilities.

## Methods

### Strain and plasmid selection

*E*. *coli* strain MG1655 was used for growth/biofilm characterisation experiments. Fluorescent protein experiments utilised BL21(DE3) *E*. *coli*, apart from the optogenetics experiments that utilised BW29655. Bacterial strains were made chemically competent by treatment with calcium chloride and transformations were performed via heat shock. All *E*. *coli* strains used in this study are freely available (S1R Data), as are plasmids (S1S Data).

### Culture techniques

Cells were cultured in either LB (growth/biofilm experiments) or EZ rich defined media (Teknova; cat: M2105) supplemented with 1% (v/v) glycerol (for optogenetic/fluorescence experiments). All experiments were performed in 20-mL volume in 30-mL clear borosilicate glass test tubes (Fisher Scientific, 11593532) open to atmosphere. Stirring employed disc stir bars (Fisher Scientific, 11878892) at speed setting 0.6. Culture temperature was maintained at 37°C unless otherwise stated. For all fluorescent protein–measurement and optogenetic-control experiments, growing cells were maintained at an OD of 0.4 using the system's turbidostat functionality. Carbenicillin (a semisynthetic analogue of ampicillin with greater stability), chloramphenicol, and spectinomycin were used at final concentrations of 100 μg mL^−1^, 25 μg mL^−1^, and 50 μg mL^−1^, respectively.

### Experiment setup procedure

Prior to experiments, test tubes and stir bars were sterilised by autoclave. Silicone tubing was sterilised by pumping 70% ethanol for 10 seconds and media for 5 seconds. For experiments that required chemical induction (Application 3), an additional 500 mL of tap water was pumped through each tube prior to the experiment to prevent inducer cross contamination. Tubing exterior was sterilised via swabbing with 70% ethanol. For each media type, each reactor's OD zero point was calibrated with a test tube of fresh media as described in S1E Data. Test tubes were filled with 20 mL media and inoculated prior to insertion into each reactor. All experiments were run directly through the Chi.Bio web interface, which was supplemented with ‘custom programs’ where indicated.

### Biofilm experiments

In each trial, cells were initially diluted to an OD of 0.1 and (once reached) maintained at an OD of 0.5. Each experiment's zero-time is defined as the time when the culture first reached OD 0.5 (typically about 2 hours post inoculation). To develop a biofilm-forming phenotype, *E*. *coli* was grown at high density (OD >1) in LB media in a reactor in chemostat mode for 120 hours. Samples from this culture were subsequently used to inoculate each of the ‘adapted’ biofilm trials.

### Fluorescence measurements

GFP was excited by the 457-nm LED at power setting 0.1 and measured at ×512 gain and 0.7-second integration time using the Clear filter as base-band and 550-nm filter as emission band. RFP was excited by the 595-nm LED at power setting 0.1 and measured at ×512 gain and 0.7-second integration time using the Clear filter as base-band and 670-nm filter as emission band. A baseline fluorescence (corresponding to the fluorescence measured for wild-type cells without plasmid) was subtracted from each measurement. Measurements were then smoothed using a moving mean filter with 10-minute width. Examples of raw (unprocessed) data and a detailed analysis of the fluorescence measurement procedure are presented in S1P Data.

### Optogenetic control

In optogenetic experiments that required full activation/deactivation (Fig 4A and 4B), either the green (525 nm) or red (625nm) LED was enabled individually at power setting 0.1. In experiments in which the precise induction level was controlled (Fig 4C and 4D), the procedure suggested by Olson and colleagues [5] was followed; the red LED was illuminated at a fixed power setting of 0.1, and the green LED's intensity was varied.

### Chemically induced reporters

In fluorescence experiments that used chemically inducible fluorescent reporters, L-Rhamnose concentrations of 0.1 mg mL^−1^ and aTc concentrations of 300 nM were used. At the time of induction, each inducer was added manually to both the reaction chamber and the fresh media supply (to provide a sharp step increase) without stopping the experiment.

## Supporting information

S1 Data Detailed design and characterisation data for Chi.Bio, and experimental notes. **S1A Data,** Electrical Architecture of Chi.Bio Platform. **S1B Data,** Control Computer. **S1C Data,** Main Reactor. **S1D Data,** Pump Board. **S1E Data,** OD Measurement and Calibration. **S1F Data,** Laser Analysis. **S1G Data,** LED/Spectral Analysis. **S1H Data,** Stirring Analysis. **S1I Data,** Operating System. **S1J Data,** User Interface. **S1K Data,** Customisation. **S1L Data,** Temperature Regulation. **S1M Data,** OD Regulation. **S1N Data,** UV Recovery. **S1O Data,** Biofilm Measurements. **S1P Data,** Fluorescence Measurements. **S1Q Data,** Other Applications. **S1R Data,** Strains Used. **S1S Data,** Plasmids Used. (PDF)
(PDF)

## Abbreviations

a.u.: arbitrary units
CL: closed loop
GFP: green fluorescent protein
LED: light-emitting diode
MPC: model predictive control
OD: optical density
PC: personal computer
PCB: printed circuit board
PI: proportional-integral
PID: proportional-integral-derivative
PWM: pulse-width modulation
RFP: red fluorescent protein
